# Non-Pharmacological Interventions for Caregivers of People with Motor Neurone Disease: A Scoping Review of Psychosocial Outcomes

**DOI:** 10.3390/brainsci15020112

**Published:** 2025-01-24

**Authors:** Chidera Okoh, Leighanne Mayall, Selina M. Makin, Cliff Chen, Nicolò Zarotti

**Affiliations:** 1 Department of Clinical Neuropsychology, Manchester Centre for Clinical Neurosciences, Salford Royal Hospital, Salford M6 8HD, UK; 2 Department of Clinical Neuropsychology, Walton Centre NHS Foundation Trust, Liverpool L9 7LJ, UK; 3 Division of Health Research, Faculty of Health and Medicine, Lancaster University, Lancaster LA1 4YW, UK

**Keywords:** motor neurone disease, amyotrophic lateral sclerosis, caregivers, caregiving, clinical psychology, psychosocial, non-pharmacological interventions, outcome

## Abstract

**Objective**: Caregivers of individuals with motor neurone disease (MND) face a wide range of psychosocial difficulties. To address these, non-pharmacological interventions have been trialled, showing promising results. However, no clear characterisation of the breadth of psychosocial constructs examined by the interventions is currently available, resulting in the lack of a core outcome set (COS). The present review explored the types of psychosocial outcomes investigated in studies that adopted non-pharmacological interventions with caregivers of people with MND. **Methods**: A scoping review was conducted across four major databases (Academic Search Ultimate, CINAHL, PsycINFO, and MEDLINE) from inception to the 1 March 2024. **Results**: From an initial return of 4802 citations, 10 were considered eligible for inclusion. A total of 10 main psychosocial outcomes were identified: anxiety and depression, psychological distress, resilience, caregiver burden, caregiver preparedness, self-efficacy, quality of life, spiritual wellbeing, and mindfulness. **Conclusions**: Caregiver burden and symptoms of anxiety and depression represent pivotal outcomes, but caution is advised with regard to caregiver burden’s potential multidimensional structure. Psychological distress and quality of life are also commonly investigated, but clearer consensus is needed on their conceptualisation. There is a paucity of studies characterising important psychosocial outcomes such as resilience, problem-solving, self-efficacy, and mindfulness, while no investigations are available for relevant outcomes such as coping, isolation, and loneliness. Further research is warranted to address these gaps to improve our insight into non-pharmacological support for MND caregivers and ultimately lead to the development of a core psychosocial outcome set in this population.

## 1. Introduction

Motor neurone disease (MND), also known as amyotrophic lateral sclerosis (ALS), is a progressive neurological disorder characterised by the degeneration of upper and/or lower motor neurones. It causes progressive paralysis and consequent difficulties with movement, speech, swallowing, and breathing [1,2] and is considered to be on a spectrum with frontotemporal dementia (FTD) [3,4]. Psychological difficulties are common in people with MND (pwMND), with evidence indicating that up to 44% of individuals experience depression and up to 30% experience anxiety directly attributed to their experience of living with MND [5]. Currently, no cure is available for MND, and palliative care represents the mainstay of its clinical management [6].

As the disease progresses, pwMND often face increasing struggles with mobility, personal care, and other activities of daily living (ADLs), ultimately leading to a need for continuous care [7]. This is frequently provided by informal caregivers, namely family members or friends [8], and involves support with ADLs such as eating, bathing, taking medication, and mobilising [9]. These can exert a significant toll on caregivers’ wellbeing, physically (e.g., moving wheelchairs or hoisting and transferring) and emotionally (e.g., witnessing deterioration in their loved one or discussing end-of-life care) [10,11]. Many caregivers experience marked role changes related to carrying out activities that were once the responsibility of their partner/relative (e.g., cooking, driving) [10,12]. Moreover, since the progression of MND can be rapid and unpredictable [13,14], caregivers often need to adjust to changes within a very short period of time, leaving them feeling unable to live their lives as before [15].

As such, caregivers frequently face psychosocial difficulties [10,16], defined as issues produced by social, occupational, or environmental circumstances (e.g., isolation) that affect psychological wellbeing [17]. Indeed, MND caregivers often experience high levels of depression, anxiety, and grief [18], as well as reduced communication, increased loneliness and helplessness, and decreased sense of self [19,20]. These issues have been shown to be more severe in caregivers of pwMND than those for other neurodegenerative diseases, such as Parkinson’s disease and multiple sclerosis [21]. Caregiver wellbeing is often crucial for the wellbeing of pwMND since bidirectional relationships between the emotional wellbeing of both parties have been identified [9,22]. Additionally, caregiving capacity can be a deciding factor in whether pwMND remain at home or enter a care facility [9,10,16,17,18,19,20,21,22,23].

To address psychosocial difficulties, non-pharmacological interventions have been trialled with MND caregivers. For instance, a mixed-methods systematic review [24] identified several interventions targeting psychological wellbeing in caregivers of pwMND, including self-management, mindfulness, dignity therapy, cognitive behavioural therapy (CBT), and person-centred therapy. The results found that, while a few studies yielded promising results with regard to psychosocial outcomes, a wide range of methodological issues prevented their generalisability. More recently, Oh and colleagues [25] carried out a scoping review of psychosocial interventions for both pwMND and their caregivers, identifying 25 studies exploring approaches, such as social support, mindfulness, behavioural therapy, and education programmes. Based on both quantitative and qualitative investigations, the authors concluded that the feasibility and acceptability of such interventions appear promising. However, similarly to the previous review [24], several methodological issues, such as high dropout rates, small sample sizes, and lack of randomisation, severely limited the generalisability of these findings to the overall MND caregiver population.

Whilst issues around the heterogeneity and validity of outcomes were raised by both reviews [24,25], neither provided a focused and systematic characterisation of the breadth of psychosocial constructs examined by the interventions. This represents a considerable gap in the current literature, as clarity about outcomes is a crucial part of evaluating interventions [26]. As reported with individuals affected by other chronic conditions as shown with other chronic conditions [27,28], obtaining specific insight into the outcomes adopted by previous studies can allow for the development of core outcome sets (COSs) to inform new treatment approaches.

Therefore, the overarching aim of the present review was to explore and map the range of psychosocial outcomes investigated to date in studies which adopted non-pharmacological interventions with caregivers of pwMND. More specifically, unlike the previous recent reviews [24,25], this work did not focus on the results and efficacy of non-behavioural interventions but rather addressed the types of outcomes included in these investigations and the way these were operationalised by addressing the following review question: what psychosocial outcomes have been investigated in non-pharmacological interventions for caregivers of people with motor neurone disease and with what measures?

## 2. Method

### 2.1. Design

A scoping review was conducted based on the latest guidelines by the Joanna Briggs Institute [29]. This design was adopted due to its usefulness in mapping specific elements within a body of literature characterised by paucity and heterogeneity of evidence while also retaining a systematic and replicable approach [30].

### 2.2. Inclusion Criteria

To be included, studies had to (a) be related to individuals caring informally for a person with a clinically confirmed diagnosis of MND/ALS; (b) describe the delivery of any non-pharmacological intervention where psychosocial constructs were formally assessed as primary or secondary outcomes with a quantitative measure; and (c) feature a formal comparison within-group or between-groups (e.g., with the control group). For this review, psychosocial outcomes were conceived as the target outcomes of interventions which are non-pharmacological and non-surgical in nature and designed to affect individuals’ decisions and actions decisions around their health and wellbeing [31,32]. Table 1 illustrates the PICO(S) framework for the inclusion criteria.

### 2.3. Exclusion Criteria

Studies not related to informal caregivers of people with MND, non-interventional and qualitative designs, and studies with no formal comparisons were excluded. Reviews, commentaries, letters to editors, conference proceedings, grey literature, and studies not fully published in English were also not included. These criteria were adopted to avoid including data which were irrelevant (e.g., not involving caregivers) or insufficiently characterised for the aims of this review (e.g., commentaries and conference proceedings).

### 2.4. Quality Assessment

Due to the heterogeneity of outcome conceptualisations, and in line with the latest guidance on scoping reviews [33], a formal quality appraisal of the evidence was not performed in this review. However, methodological limitations of some of the measures adopted in the included studies were discussed where relevant.

### 2.5. Procedure

**Figure 1:**
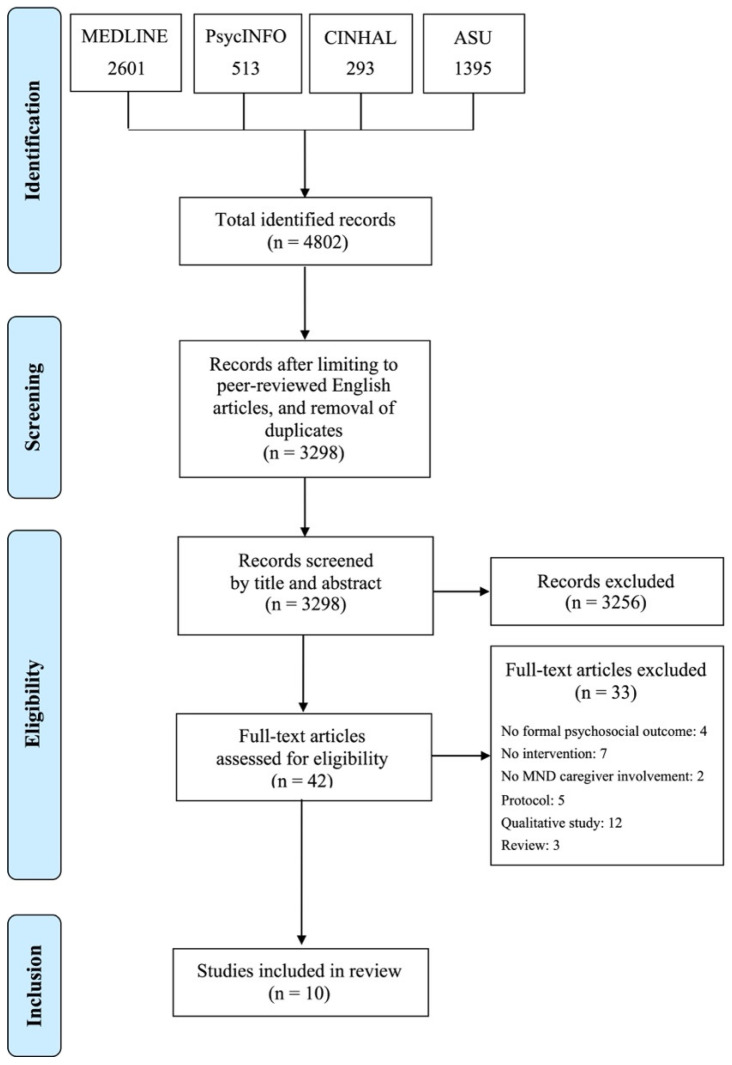
PRISMA diagram for selection of studies.

A combination of free text terms and Boolean operators were adopted to search four major databases from inception to the 1 March 2024: Academic Search Ultimate, CINAHL, PsycINFO, and MEDLINE. Reference lists of included studies were hand-searched. Table 1 shows the logic grid for the search strategy, while Table 2 illustrates the adopted search terms. Initially, two reviewers (C.O. and L.M.) screened all titles and abstracts against the inclusion criteria, which were then checked by a further three reviewers (N.Z., S.M.M., and C.C.). Following the initial screening, the full texts of the remaining citations were inspected by two reviewers (C.O. and L.M.) and confirmed by three other reviewers (N.Z., S.M.M., and C.C.). Figure 1 illustrates the PRISMA flow diagram of the study selection.

### 2.6. Protocol Registration

Since scoping reviews are currently not accepted by the international prospective register of systematic reviews (PROSPERO), no formal registration was carried out for the protocol of the present review.

## 3. Results

The database searches yielded a total of 4802 citations, which was reduced to 3298 citations following de-duplication. Screening based on title and abstract led to the exclusion of 3261 papers, leaving 37 full-text citations to be considered for inclusion. After full-text screening, 10 papers were considered eligible for the review. Table 3 illustrates the main characteristics of the included studies. The list of citations excluded following full-text screening is available as Supplementary Materials. Due to this review’s specific focus on the breadth, conceptualisation, and measurement of outcomes, the characteristics of the participants, as well as the results of the interventions in terms of efficacy in each study, are not reported below. However, a brief summary of these has been included in Table 4 to help contextualise the present findings.

Of the ten included studies, six were interventions designed specifically for MND caregivers [34,35,36,37,38,39], whereas four studies included both MND patients and caregivers [40,41,42,43]. The outcomes investigated by the interventions are reported below. Higher levels of evidence hierarchy, such as randomised controlled trials (RCTs), are highlighted when appropriate.

### 3.1. Anxiety and Depression Symptoms

Due to the high rate of co-investigation of symptoms of depression and anxiety both as constructs as well as across single measure tools (e.g., HADS), these have been grouped together in the present review. 

Five studies investigated symptoms of anxiety and depression in MND caregivers [34,36,38,40,42]. Two of these [36,42] were RCTs. The Hospital Anxiety and Depression Scale (HADS) [44] was the most adopted measure, featuring four studies [34,36,40,42]. The remaining citation [38] adopted the Brief Profile of Mood States (POMS) [45] and the 10-item Centre for Epidemiology Studies Depression Scale (CES–D) [46].

### 3.2. Psychological Distress

Psychological distress constructs—such as grief, hopelessness, and stress—were investigated by four studies [34,37,38,39]. Of these, one was an RCT [37], which adopted the Italian version of the Perceived Stress Scale (PSS) [47]. The remaining three citations, all pre–post designs [34,38,39], adopted the Brief Symptom Inventory 18 (BSI-18) [48], the Herth Hope Index (HHI) [49], and the 12-item Prolonged Grief Scale (PG-12) [50], which assesses feelings of grief leading up to an expected death.

### 3.3. Resilience

One RCT [37] investigated resilience in MND caregivers adopting the Connor Davidson Resilience Scale (CD-RISC) [51], a measure which explores an individual’s ability to bounce back following adverse life events.

### 3.4. Caregiver Burden

Caregiver burden, which has been defined as the “level of multifaceted strain perceived by the caregiver from caring for a family member and/or loved one over time” [52], was investigated as an outcome by nine studies [34,36,37,38,39,40,41,42,43]. Of these, five [36,37,41,42,43] were RCTs. The most frequently adopted measure was the Zarit Burden Interview (ZBI) [53], featuring four studies [34,36,40,42]. Other adopted measures included the Caregiver Reaction Assessment (CRA) [54], the Caregiver Strain Index (CSI) [55], and the Caregiver Burden Inventory (CBI) [56].

### 3.5. Caregiver Preparedness

Caregiver preparedness was investigated by one study based on a pre–post design [39] and adopting the Preparedness for Caregiving Scale [57].

### 3.6. Problem-Solving

A pilot pre–post study [39] explored caregivers’ perceived problem-solving skills with the Problem Solving Inventory (PSI) [58].

### 3.7. Self-Efficacy

One pre–post study [35] adopted exploratory ad hoc items to assess caregivers’ self-efficacy and feelings of goal attainment across a range of different caregiving activities (e.g., meal preparation, toileting, and administering medication) measured with a modified young carers version of the Multidimensional Assessment of Caring Activities (MACA-YC18).

### 3.8. Quality of Life

The quality of life of MND caregivers was investigated as an outcome of four studies [36,38,42,43]. These included three RCTs [36,42,43], which adopted the Mental Component Summary Score of the Medical Outcomes Study Questionnaire Short Form 36 (SF-36-MCS) [59]. Other measures included the McGill’s Quality of Life Questionnaire [60] and the Quality of Life at the End of Life (QUAL-E) [61].

### 3.9. Spiritual Wellbeing

Caregivers’ spiritual wellbeing was explored as an outcome in a pre–post study [38] using the Functional Assessment of Chronic Illness Therapy-Spiritual Well-Being (FACIT-Sp), which measures a sense of meaning and the role of faith in experiencing a long-term illness [62], and the Brief Religious Coping Activity Scales (RCOPE) [63], which focuses on positive and negative religious coping styles.

### 3.10. Mindfulness

Only one pre–post study [39] investigated caregivers’ levels of mindfulness by using the Cognitive and Affective Mindfulness Scale—Revised (CAMS-R) [64].

## 4. Discussion

### 4.1. Summary of Main Findings

This scoping review aimed to map the breadth of psychosocial outcomes which have been investigated by non-pharmacological interventions involving caregivers of pwMND. To our knowledge, this is the first review to explore this area. From an initial return of 4802 citations, 10 were eventually considered eligible for inclusion in the review. Half of these studies were RCTs [36,37,41,42,43], while the other five adopted a pre–post design [34,35,38,39,40]. A total of 10 main psychosocial outcomes were identified in the included studies: anxiety and depression symptoms, psychological distress, resilience, caregiver burden, caregiver preparedness, problem-solving, self-efficacy, quality of life, spiritual wellbeing, and mindfulness.

Caregiver burden was the most frequent outcome, being investigated by nine studies out of ten [34,36,37,38,39,40,41,42,43]. This was mostly measured with the ZBI [53], a self-report questionnaire with a long history of adoption not only with caregivers of individuals with MND [8] but also with other neurodegenerative conditions such as Alzheimer’s [65,66], Parkinson’s [67,68], and Huntington’s [69,70]. However, a factor analysis of the ZBI carried out with MND family caregivers highlighted a 3-factor structure encompassing social restrictions, self-criticism, and anger and frustration [71]—suggesting that interventions may need to consider a multifactorial view of caregiver burden when planning interventions in this population.

The second most frequent psychosocial outcome was represented by symptoms of anxiety and depression, which were investigated by half of the included studies [34,36,38,39,40]. This is perhaps unsurprising since these issues have been shown to be prevalent among MND caregivers [72,73] and related to increased burden, lower quality of life, and higher risk of health problems [74,75]. The most adopted measure of anxiety and depression symptoms was the Hospital Anxiety and Depression Scale (HADS) [44]—a finding that mirrors a previous systematic review which identified the HADS among the most frequent measures across cross-sectional and longitudinal studies of caregiver burden in MND [8].

Four studies investigated psychological distress [34,37,38,39] and quality of life [36,38,42,43], making them the third most common psychosocial outcome. Similarly to symptoms of anxiety and depression, both constructs have often been the focus of studies involving MND caregivers [75,76,77]. However, the wide range of measures observed in this review suggests that their conceptualisation may vary considerably across researchers, with some authors focusing on specific aspects such as grief, hopelessness, or end of life [34,38,40], while others explored more general forms of stress or quality of life [36,39,42,43].

The remaining six psychosocial outcomes—resilience, caregiver preparedness, problem-solving, self-efficacy, spiritual wellbeing, and mindfulness—were investigated only by one study each. While constructs such as spirituality have not frequently been the object of exploration in MND caregivers [78], the paucity of studies investigating resilience, mindfulness, problem-solving, and self-efficacy as intervention outcomes, represents a more unexpected finding, particularly considering the significant attention they have received in non-interventional studies with this population [79,80,81,82,83,84]. Perhaps even more surprising was that one of the two mindfulness-based interventions in this review did not include a measure of mindfulness among its outcomes [36]. In addition, no studies were identified for other psychosocial outcomes such as (non-religious) coping, isolation, and loneliness—all of which have been previously highlighted as pivotal in MND caregivers [85,86,87].

### 4.2. Implications for Future Interventions

The findings of the present review show a number of important implications for the future development of non-pharmacological interventions for MND caregivers, and the conceptualisation and evaluation of a future core psychosocial outcome set. First, caregiver burden and difficulties related to anxiety and depression were identified as the most common outcomes across all studies, highlighting that their operationalisation is likely to represent a crucial part of an outcome set. However, caution should be advised when operationalising caregiver burden as a unifactorial construct, as evidence has suggested a potential multidimensional structure in widely adopted measures such as the ZBI. Thus, future studies should aim to explore this outcome in a more multifaceted fashion, potentially adopting a more comprehensive set of measures.

Secondly, while the findings of this review highlighted psychological distress and quality of life as important outcomes for non-pharmacological interventions for MND caregivers, the high level of variability in how such constructs are conceptualised by researchers (as well as caregivers themselves) should be considered. Therefore, a clearer consensus is needed on the operationalisation of psychological distress and quality of life in this population, and future interventions would benefit from including more comprehensive descriptions and rationales for adopting specific measures.

Finally, our findings highlighted a severe paucity of intervention studies assessing important psychosocial outcomes such as resilience, problem-solving, self-efficacy, and mindfulness, even when they represented the core of specific interventions. Further investigations are therefore warranted to address these in MND caregivers, along with other relevant outcomes for which no studies were identified in the present review, such as coping, isolation, and loneliness.

### 4.3. Limitations

A number of limitations should be considered with the present findings. First, while they allow to map of emerging studies characterised by a paucity of studies, scoping reviews provide preliminary overviews which preclude more definite clinical recommendations and should thus be followed by systematic reviews as additional evidence accrues. Secondly, issues such as conceptual diversity and overlapping for some constructs and outcomes (e.g., psychological distress and quality of life) represent a further limitation and future studies should aim to work towards reaching a consensus on this matter. Finally, all the eligible studies in this review were carried out in Western countries and may limit the value of the implications of this review across different settings. Further investigations are therefore needed involving MND caregivers in under-represented countries and cultures.

## 5. Conclusions

This scoping review shed new light on the current conceptualisation and operationalisation of psychosocial outcomes in non-pharmacological interventions for MND caregivers. Caregiver burden symptoms of anxiety and depression appear to be pivotal outcomes, but caregiver burden should be explored as a multifaceted construct. Psychological distress and quality of life are also important, but their conceptualisation varies greatly, necessitating a clearer consensus among researchers. Nonetheless, important psychosocial outcomes such as resilience, problem-solving, self-efficacy, and mindfulness have received considerably less attention in this population. Further intervention studies are warranted to address these gaps and consider other relevant outcomes such as coping, isolation, and loneliness to improve our insight into non-pharmacological support for MND caregivers and ultimately lead to the development of a core psychosocial outcome set.

## Figures and Tables

**Table 1:** PICO(S) framework.

Population	Interventions	Comparison	Outcome	Study
Informal caregivers of individuals with motor neurone disease	Non-pharmacological and non-surgical interventions designed to affect actions and decisions around health and wellbeing	Within-group or between-groups (e.g., control)	Psychosocial constructs formally assessed as primary or secondary outcomes	Cross-sectional or longitudinal studies adopting quantitative methodologies

**Table 2:** Logic grid for the search strategy.

Population Terms	Intervention Terms	
Motor neuron* disease Amyotrophic lateral sclerosis Caregiver care Carer Family Next of kin Relative Spouse Child Parent	Acceptance and commitment therapy Activity Behavio* therapy Cognitive analytic therapy Cognitive behavio* therapy Cognitive therapy Compassion* focused therapy Counsel* Couple* therapy Dialectical behavioral therapy Emotion focused therapy Exercise Emotive behavio* therapy Eye movement desensiti* and reprocessing Family therapy Gestalt therapy Group* therapy Integrative therapy Interpersonal therapy Intervention Learning Lifestyle Meditat*	Metacognitive therapy Mindfulness Mindfulness-based cognitive therapy Mindfulness-based stress reduction Motivational interviewing Narrative therapy Person cent* therapy Program* Psychoanal* Psychodynamic therapy Psychoeducati* Psychological intervention Psychosocial intervention Psychotherap* Rational emotive behavio* therapy Rehabilitation Schema therapy Self-management Solution focused therapy Support Systemic therapy

**Table 3:** Adopted search terms.

Search Terms	Filters
(Motor neuron* disease OR Amyotrophic lateral sclerosis) AND (Caregiver care OR Carer OR Family OR Next of kin OR Relative OR Spouse OR Child OR Parent) AND (Acceptance and commitment therapy OR Behavio* therapy OR Cognitive analytic therapy OR Cognitive behavio* therapy OR Cognitive therapy OR Compassion* focused therapy OR Counsel* OR Couple* therapy OR Dialectical behavioral therapy OR Emotion focused therapy OR Emotive behavio* therapy OR Eye movement desensiti* and reprocessing OR Family therapy OR Gestalt therapy OR Group* therapy OR Integrative therapy OR Interpersonal therapy OR Meditat* OR Metacognitive therapy OR Mindfulness OR Mindfulness-based cognitive therapy OR Mindfulness-based stress reduction OR Motivational interviewing OR Narrative therapy OR Person cent* therapy OR Psychoanal* OR Psychodynamic therapy OR Psychoeducati* OR Psychological intervention OR Psychotherap* OR Rational emotive behavio* therapy OR Schema therapy OR Self-management OR Solution focused therapy OR Systemic therapy OR Intervention OR Psychosocial intervention OR Program* OR Rehabilitation OR Activity OR Lifestyle OR Support OR Exercise OR Learning)	English language
	Human participants
	Boolean/Phrase
	Subject/related terms

**Table 4:** Key characteristics of included studies.

Study	Country	Design	Sample	Intervention	Relevant Outcomes	Relevant Measures	Key Results
[34]	Australia	Pre–Post	I: 18	Dignity Therapy	Caregiver burden Psychological distress Anxiety Depression	ZBI-22 HADS HHI	No significant changes at the group level but significant decreases in anxiety and depression at the individual level.
[35]	USA	Pre–Post	I: 19	YCare training and support protocol	Self-efficacy	MACA-YC18 Ad hoc exploratory items	Significant increase in confidence in tasks.
[36]	USA	RCT	I: 11 C: 11	Mindfulness	Quality of life Anxiety Depression Caregiver burden	QoL-SF36 HADS ZBI-22	Significant improvements in depression, anxiety, care burden scores, and specific elements of quality of life in the intervention group compared to controls.
[37]	Italy	RCT	I: 6 C: 6	Individual tele-consults and resilience-oriented sessions of group therapy	Caregiver burden Resilience Psychological distress	CBI CD-RISC PSS	No significant changes.
[38]	USA	Pre–Post	I: 9	Chaplain-led intervention sessions	Quality of life Spiritual wellbeing Caregiver burden Psychological distress Depression	QUAL–E FACIT–Sp POMS CES–D PG-12 RCOPE CRA	No clinical levels of psychosocial difficulties at baseline, and outcomes remained stable over time.
[39]	Australia	Pre–Post	I: 13	Caregiver self-care, problem-solving and mindfulness	Caregiver burden Caregiver preparedness Mindfulness Problem-solving	BSI-18 CRI PSI PCS CAMS	No significant changes.
[40]	Australia	Pre–Post	I: 18 P: 25	Dignity Therapy	Caregiver burden Anxiety Depression Hopefulness	ZBI-22 HADS HHI	No significant changes.
[41]	Netherlands	RCT	I: 66 P: 71 CC: 60 CP: 61	Case Management	Caregiver burden	CSI	No significant changes.
[42]	Netherlands	RCT	I: 74 C: 74	Blended psychosocial support programme based on an ACT approach	Psychological distress Caregiver burden Quality of life	HADS ZBI-22 CRQoL MQOL	No significant changes.
[43]	Netherlands	RCT	I: 10 C: 5	CBT	Quality of life Psychological distress Caregiver burden	SF-36-MCS HADS CSI	Significantly better quality of life caregiver burden in the intervention group.

Note. ACT: acceptance and commitment therapy; BSI-18: Brief Symptom Inventory; CAMS: Cognitive and Affective Mindfulness Scale; CBT: cognitive behaviour therapy; C: control; CC: control caregivers; CBI: caregiver burden inventory; CD-RISC: Connor Davidson resilience scale; CES–D: 10-item Centre for Epidemiology Studies Depression Scale; CPs: control patients; CRA: Caregiver Reaction Assessment; CRI: Caregiver Reaction Assessment; CRQoL: Care Related-Quality of Life questionnaire; CSI: caregiver Strain Index; FACIT–Sp: functional assessment of chronic illness therapy—spiritual wellbeing; MACA-YC18: Multidimensional Assessment of Caring Activities; MQOL: McGill Quality of Life Questionnaire; QUAL–E: Quality of life at the end of life; QoC: Quality of Care; PCS: Preparedness for Caregiving Scale; PSI: Problem Solving Inventory; I: intervention; P: patient; PG-12: 12-item Prolonged Grief Scale; POMS: Brief Profile of Mood States; PSS: Perceived stress scale; RCT: randomised control trial; RCOPE: Brief Religious Coping Activity Scales; ZBI-22: Zarit Burden Interview.

## Supplementary Materials

The following supporting information can be downloaded at: https://www.mdpi.com/article/10.3390/brainsci15020112/s1, Preferred Reporting Items for Systematic reviews and Meta-Analyses extension for Scoping Reviews (PRISMA-ScR) Checklist. Reference [88] is cited in Supplementary Materials.
